# Exercise economy in skiing and running

**DOI:** 10.3389/fphys.2014.00005

**Published:** 2014-01-24

**Authors:** Thomas Losnegard, Daniela Schäfer, Jostein Hallén

**Affiliations:** Department of physical performance, Norwegian School of Sport SciencesOslo, Norway

**Keywords:** cross-country skiing, cross country skiers, inter-individual variations, intra-individual variations, running

## Abstract

Substantial inter-individual variations in exercise economy exist even in highly trained endurance athletes. The variation is believed to be determined partly by intrinsic factors. Therefore, in the present study, we compared exercise economy in V2-skating, double poling, and uphill running. Ten highly trained male cross-country skiers (23 ± 3 years, 180 ± 6 cm, 75 ± 8 kg, VO_2peak_ running: 76.3 ± 5.6 mL·kg^−1^·min^−1^) participated in the study. Exercise economy and VO_2peak_ during treadmill running, ski skating (V2 technique) and double poling were compared based on correlation analysis. There was a very large correlation in exercise economy between V2-skating and double poling (*r* = 0.81) and large correlations between V2-skating and running (*r* = 0.53) and double poling and running (*r* = 0.58). There were trivial to moderate correlations between exercise economy and the intrinsic factors VO_2peak_ (*r* = 0.00–0.23), cycle rate (*r* = 0.03–0.46), body mass (*r* = −0.09–0.46) and body height (*r* = 0.11–0.36). In conclusion, the inter-individual variation in exercise economy could be explained only moderately by differences in VO_2peak_, body mass and body height. Apparently other intrinsic factors contribute to the variation in exercise economy between highly trained subjects.

## Introduction

The speed achieved in endurance competitions depends on several physiological and mechanical factors. One of these factors is exercise economy, defined as the amount of energy spent per unit of velocity (di Prampero, 2003), and there have been reports of close relationships between exercise economy and performance in several endurance sports (Conley and Krahenbuhl, 1980; Saunders et al., 2004). While it is known that exercise economy is quite variable between athletes (e.g., Coyle et al., 1992; Saunders et al., 2004; Losnegard et al., 2012), the sources of inter-individual variance are not well understood. Therefore, factors which may explain this phenomenon in running and cycling have been thoroughly investigated (Coyle et al., 1992; Saunders et al., 2004; Lucia et al., 2006; Ettema and Lorås, 2009).

Coyle et al. (1992) found a strong correlation between fibertype 1 and exercise economy both in cycling and two-leg extension exercise. Furthermore, Lucia et al. (2006) showed that the best Eritrean runners had a significantly better exercise economy, despite a lower training experience and volume, than their Spanish counterparts. These differences in exercise economy are probably more related to anthropometrics (long, slender shanks, and lower body mass index) rather than metabolic differences (Saltin et al., 1995; Lucia et al., 2006). In complex techniques as ski-skating, Losnegard et al. (2012) showed that there was a large inter-individual variance in exercise economy, but a relatively low intra-individual variance in elite cross-country skiers performing two different ski skating techniques. Thus, subjects who were most economical in one ski skating technique were also relatively economical in the other ski skating technique, even if these two techniques are shown to be biomechanically very different (Myklebust et al., 2013). These studies indicate that intrinsic factors contribute significantly to the variance in exercise economy between highly trained subjects.

Based on these notions, we hypothesized that the most economical subjects in one type of cyclic exercise also are the most economical in another cyclic exercise as long as the legs are a main contributor to the energy expenditure. In this context, highly trained cross-country skiers serve as a unique group of subjects, as they perform a large and similar amount of training in running (~25% of total endurance training), ski skating (~25%), and in the classical skiing style (~25%) (Losnegard et al., 2013).

Hence, in the present study, we compared exercise economy in V2-skating, double poling, and uphill running. In all these exercise modes the legs are the main energy consumer even if the arms are involved in the propulsion in the skiing techniques, more in double poling than V2-skating technique (Calbet et al., 2005; Smith et al., 2009; Rud et al., 2013).

## Materials and methods

### Subjects

Ten male cross-country skiers (age: 23 ± 3 years, height: 180 ± 6 cm, body mass: 75 ± 8 kg) volunteered to participate in this project. All subjects had 10–15 years of cross-country skiing training background and four subjects were national elite-level skiers. Inclusion criteria were a peak oxygen uptake >65 mL·kg^−1^·min^−1^ (running test). The study was approved by the Regional Ethics Committee of Southern Norway and the subjects gave their written consent to participate.

### General overview

**Figure 1:**
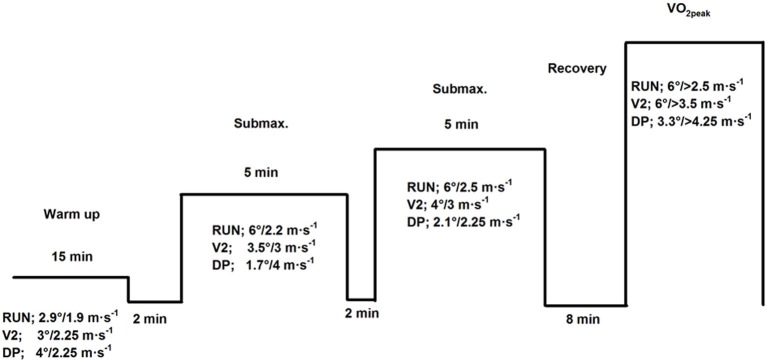
**Schematic protocol of the study**. Steady-state VO_2_ (exercise economy) was measured at two submaximal workloads of 5 min each, followed by a maximal test performed as a 1000-m test in the two skiing techniques and an incremental test in running. Exercise modes were V2-skating, double poling and running.

After two to four training familiarization sessions, the subjects performed submaximal trials and a VO_2peak_ test in V2-skating, double poling, and running in a randomized order (Figure 1). All tests were performed on a treadmill (rollerski for the skiing techniques) and were completed within 2 weeks, with at least one day between two consecutive tests.

### Exercise economy

Prior to testing, the subjects warmed-up for 15 min at an incline/speed of 2.9°/1.9 m·s^−1^ (running), 3°/2.25 m·s^−1^ (V2-skating), and 4°/2.25 m·s^−1^ (double poling), respectively. Double-poling warm up was performed using the diagonal stride technique. Two submaximal workloads of 5 min with a break of 2 min in between were conducted (Figure 1). The workloads for the different exercise modes were chosen to achieve nearly the same average % of VO_2peak_ in the different exercise modes (based on pilot and training sessions). For all subjects, the speed was 3 m·s^−1^ with inclines of 3.5° and 4° in V2-skating while during double poling the speed was 4 m·s^−1^ with inclines of 1.7° and 2.1°. In running the incline was 6° and speed 2.2 and 2.5 m·s^−1^. All data are presented as an average of the two submaximal stages. After 4 min at each workload, the subjects determined their rating of perceived exertion (RPE) which was reported in the breaks between workloads. Exercise economy was defined as the average oxygen uptake (VO_2_) relative to body mass (mL·kg^−1^·min^−1^) between 3 and 4.5 min during the submaximal workloads. Other physiological parameters such as heart rate (HR), ventilation (VE), and respiratory exchange ratio (RER) were also averaged over the same time span. Immediately after the 5-min submaximal trial and after the VO_2peak_ tests, blood lactate concentration was measured in unhemolyzed blood collected from capillary fingertip samples. We also calculated the gross efficiency in V2-skating and DP to check the correlation between different methods for analyzing economy. Aerobic energy turnover rate (Watts) was calculated using the VO_2_ and the corresponding RER (<1.0) (Foss and Hallén, 2005). External power was calculated as the sum of the power against gravity and the power against rolling friction (Losnegard et al., 2013). Gross efficiency was defined as the ratio between external power output (Watts) and aerobic energy turnover rate (Watts) and expressed in percentage units.

### Kinematic variables

Cycle rate and cycle length were determined by video analysis with Dartfish Connect 4.5 (Dartfish LTD., Fribourg, Switzerland). One cycle was defined as the time between two consecutive belt contacts of the right shoe or ski in running and V2-skating and between two pole plants of the right pole in double poling. The average of 10 consecutive cycles, starting at 3 min of each submaximal workload, was used for the analysis.

### Peak oxygen uptake and performance

The VO_2peak_ tests in V2-skating and double poling were conducted 8 min after the last submaximal trial as a 1000-m time trial, where the goal was to ski as fast as possible (Losnegard et al., 2013). The incline remained constant at 3.3° for double poling and 6° for V2-skating. To avoid over-pacing, the speed was standardized at 3.25 and 3.5 m·s^−1^ for the first and second 100 m, respectively, in V2-skating and at 4 and 4.25 m·s^−1^ in double poling. Afterwards, the skiers could regulate their speed by leaving the zone between two laser markers. The speed was increased when the skier's front wheel passed the laser mark in front or decreased when the skier dropped behind the rear mark. VO_2peak_ in running was performed at an incline of 6°. The test started at individual speed (2.5–3 m·s^−1^) according to the skier's running level and was increased incrementally by 0.28 m·s^−1^ each min until exhaustion. Exhaustion occurred within 4.5–6.5 min. This test has been used for decades in our lab, and shows high reliability for cross-country skiers (coefficient of variation; *CV* = 2.2%). During all tests, oxygen consumption was measured continuously in 5 s intervals and the highest oxygen value averaged over 60 s was considered as the exercise specific VO_2peak_.

### Measuring apparatus

Oxygen consumption was measured by an automatic ergospirometry system (Oxycon Pro, Jaeger Instrument, Hoechberg, Germany), which was evaluated by Foss and Hallén (2005). Heart rate was measured with a Polar S610i monitor (Polar Electro Oy, Kempele, Finland), and blood lactate concentration was measured in unhemolyzed blood from capillary fingertip samples (YSI 1500 Sport; Yellow Springs Instruments, Yellow Springs, OH, USA). The lactate analyzer and Oxycon Pro were calibrated according to the instruction manual, as described in detail previously (Losnegard et al., 2011). Tests were performed on a rollerski treadmill (Rodby, Sodertalje, Sweden) with dimensions of 3 × 4.5 m and on a running treadmill (Woodway GmbG, Weil am Rein, Germany). Incline and speed accuracy were controlled continuously. During the VO_2peak_ skiing tests, the skiers were secured with a safety harness connected to an automatic emergency brake system. Two identical pairs of classic and skating rollerskis (Swenor, Sarpsborg, Norway) with type I wheels for ski skating and type II (front wheel) and type III wheels (rear wheel) for classic skis were used with two different binding systems (NNN, Rottefella, Klokkarstua, Norway or SNS, Salomon, Annecy, France). For each athlete, the most familiar binding system was used. The rolling friction of the rollerskis was regularly controlled during the whole testing period, following the instructions outlined by Hoffman et al. (1990). The standardized warm-up of 15 min avoided a warm-up effect of the wheels and other mechanical parts during the test session. The rolling friction coefficient was 0.020 and 0.026 for skating and classic rollerskis, respectively. Swix CT1 poles (Swix, Lillehammer, Norway) with customized tips for treadmill rollerskiing were used. The pole length was the same as normally used by the athletes and had a length of 154 ± 6 cm in double poling and 164 ± 6 in V2-skating, corresponding to 85 ± 1 and 91 ± 1% of individual body height. The subject's body mass was determined before each test (Seca, Hamburg, Germany). A stationary video camera (Sony DCR-TRV900E, Sony, Tokyo, Japan) recorded the movements of the test subjects from a lateral perspective. The camera was positioned 5 m away from the subjects.

### Statistics

All data are presented as mean ± standard deviation (SD) unless otherwise stated. Precision of estimation and magnitude-based inferences were conducted. Confidence limits (90%) for the true mean values for effects were estimated (Hopkins et al., 2009). The magnitude of differences between exercise modes was expressed as standardized mean differences (Cohen's *d* effect size; ES). The criteria to interpret the magnitude of the ES were as follows: 0.0–0.2, trivial; 0.2–0.6, small; 0.6–1.2, moderate; 1.2–2.0, large; and >2.0, very large. Pearson's product-moment correlations were used to quantify magnitude of linear relationships between variables. The intrinsic factors, body height, body mass, and VO_2peak_ were correlated to exercise economy based on studies that indicate that these variables could explain variations in exercise economy between subjects (Saunders et al., 2004; Lucia et al., 2006). Criteria for interpreting the magnitude of correlation (*r*) were: <0.1, trivial; 0.1–0.3, small; 0.3–0.5, moderate; 0.5–0.7, large; 0.7–0.9, very large; and 0.9–1.0, almost perfect (Hopkins et al., 2009). Microsoft Excel (Redmond, WA) and SigmaPlot 12.3 software (San Jose, CA) were used for statistical calculations.

## Results

### Exercise economy

**Figure 2:**
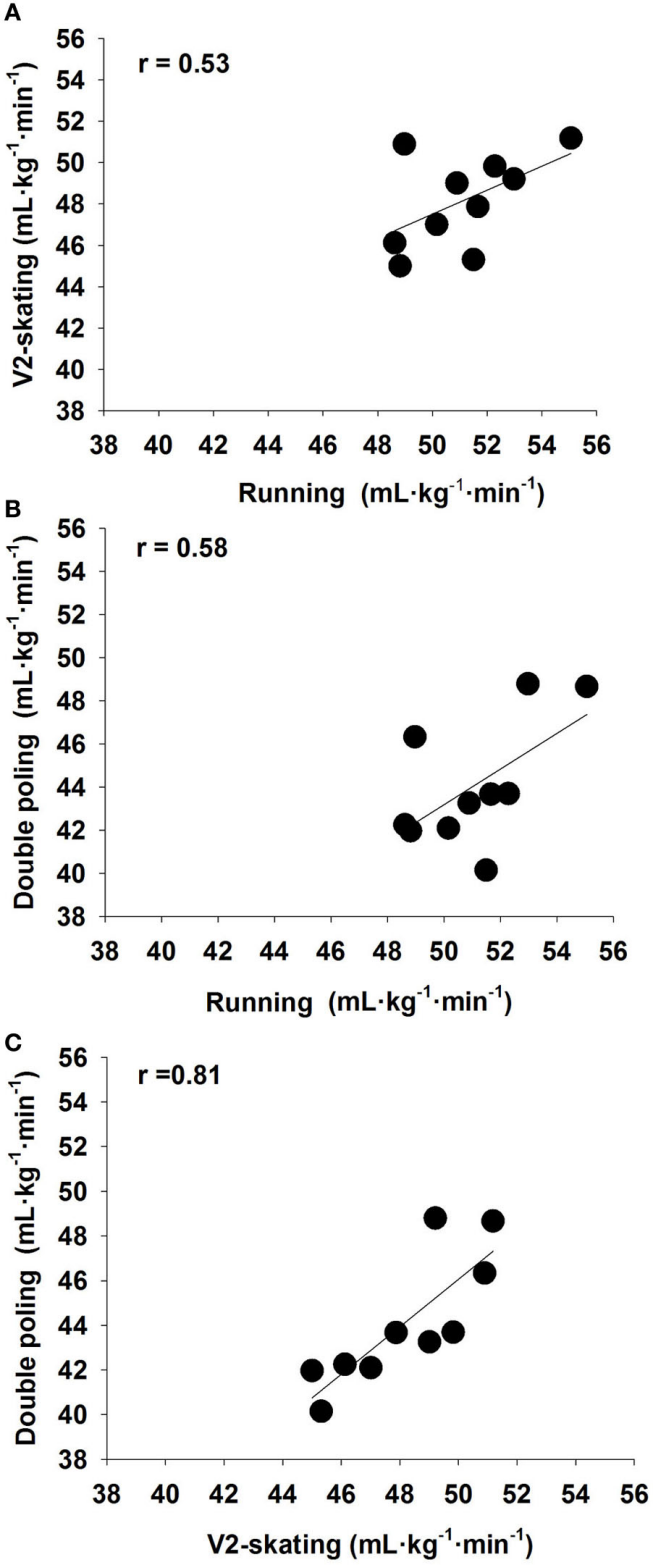
**Relation in exercise economy (oxygen cost at steady state) in running vs. V2-skating (A), running vs. double poling (B), and V2-skating vs. double poling (C) in ten male well-trained cross-country skiers**. Correlation values (*r*) were obtained using Pearson's Product Moment Correlation Analysis.

There was a very large correlation between V2-skating and double poling for exercise economy (*r* = 0.81; 90% confidence interval 0.47–0.94) and gross efficiency (*r* = 0.80; 0.44–0.94), while large correlations were observed between V2-skating and running (*r* = 0.53; −0.3–0.84) and double poling and running exercise economies (*r* = 0.58; −0.04–0.86) (Figure 2). Oxygen uptake during the submaximal loads in percentage of VO_2peak_ was similar for the three exercise modes. Ventilation and RPE were also similar between exercise modes while HR, RER, and La^−^ were higher during double poling than V2-skating and running (Table 1). Gross efficiencies in V2-skating and double poling showed a nearly perfect correlation to O_2_-cost (*r* = −0.98 for V2-skating and *r* = −0.99 for double poling).

**Table 1:** **Work load and the physiological response during submaximal and VO_2peak_ test during running and the two skiing techniques (V2-skating and double poling; *n* = 10)**.

**Variable**	**Submaximal**			**VO_2peak_**		
	**Running**	**V2-skating**	**Double poling**	**Running**	**V2-skating**	**Double poling**
Power (W)	–	198 ± 18	183 ± 17^b^			
VO_2_ (mL·kg^−1^·min^−1^)	51.1 ± 2.1	48.1 ± 2.2^c^	44.1 ± 2.9^d^	76.3 ± 5.6	72.8 ± 6.5^b^	67.0 ± 5.8^b^^c^
VO_2_ in % of VO_2peak_	67 ± 6	67 ± 7	66 ± 6			
Gross efficiency (%)	–	16.0 ± 0.7	15.9 ± 1.0			
VE (L·min^−1^)	93 ± 17	88 ± 9	91 ± 12	188 ± 18	190 ± 19	182 ± 16^a^
HR (beat·min^−1^)	155 ± 12	156 ± 15	150 ± 14^a^	190 ± 6	189 ± 6	186 ± 6^b^
HR (% of peak)	82 ± 6	82 ± 7	81 ± 8			
La^−^ (mmol·L^−1^)	1.4 ± 0.5	1.5 ± 0.8	2.7 ± 1.0^c^	7.8 ± 1.5	9.1 ± 1.0^b^	8.0 ± 1.0^a^
RER	0.88 ± 0.04	0.88 ± 0.05	0.93 ± 0.02^b^	1.08 ± 0.06	1.10 ± 0.06^a^	1.12 ± 0.08^a^^a^
RPE (6–20)	12.8 ± 1.6	12.6 ± 1.9	13.0 ± 1.5			

*Table includes the magnitude of differences (above 0.2) between exercise modes expressed as standardized mean differences (Cohen's d effect size; ES). Indicators (^a,b,c,d^) refers to column to the left. Data that includes two indicators refers to both of the other exercise modes*.

a *ES = Small (0.2–0.6)*.

b *ES = Moderate (0.6–1.2)*.

c *ES = Large (1.2–2.0)*.

d *ES = Very large (>2.0)*.

*Data are averaged values from two workloads and given as group mean ± SD. VE, Ventilation; HR, Heart rate; La^−^, blood lactate concentration; RER, Respiratory exchange ratio; RPE, Rate of perceived exertion*.

There were large inter-individual differences in exercise economy with an absolute difference between the most and the least economical skiers of 12% for V2-skating, 18% for double poling, and 12% for running (Figure 2). Correlations between exercise economy and 1000-m performance (finishing time) were moderate (*r* = 0.38, *r* = 0.44, V2 skating and double poling, respectively).

### Cycle rate, anthropometrics, and economy

The cycle rates at submaximal workloads were 0.50 ± 0.04 Hz for V2-skating, 0.83 ± 0.08 Hz for double poling and 1.35 ± 0.08 Hz for running. There were trivial to moderate correlations between cycle rate and exercise economy in V2-skating (*r* = −0.03), in double poling (*r* = −0.46) and in running (*r* = −0.39). Similar magnitude of correlation were found for body mass and exercise economy (*r* = −0.09 for V2-skating, *r* = −0.46 for double poling and *r* = 0.24 for running) and body height and exercise economy (*r* = 0.13 for V2, *r* = −0.11 for double poling and *r* = 0.36 for running).

### Peak oxygen uptake

VO_2peak_ was highest for running, 4.7 ± 2.6% (mean ± confidence limits) lower in V2-skating (*ES* = 0.59) and 12.3 ± 2.9% lower in double poling compared to running (*ES* = 1.62). There were very large correlations between VO_2peak_ in the different exercise modes (*r* = 0.83–0.90). There were trivial to small correlations between exercise economy and VO_2peak_ for all exercise modes (*r* = 0.00–0.23).

## Discussion

The major finding was the large to very large correlation between exercise economy in the different exercise modes running and skiing (both skating and double poling). This indicates that intrinsic factors relevant for all three exercise modes were determinants for exercise economy. Of possible intrinsic factors, body height, body mass, and VO_2peak_ could only partly explain the variations in exercise economy. Hence, other intrinsic factors, such as body-segment length, body mass and muscle-fiber type, are likely important factors determining exercise economy in all exercise modes. The non-perfect correlations are likely explained by error of measurement and different technical skills between exercise modes.

### Exercise economy relationships between subjects

The overall aim of the present study was to compare exercise economy in V2-skating, double poling, and uphill running. The study was based on the fact that exercise economy is found to vary a lot between trained subjects (Saunders et al., 2004; Lucia et al., 2006; Losnegard et al., 2012) and that this variation cannot be fully explained by training status, performance level, and technical skills. Hence, intrinsic factors unrelated to training may play a major role. These factors are for instance related to anthropometrics and muscle fiber types. A very large correlation was found in exercise economy between V2-skating and double poling while a large correlation was found between running and the two skiing techniques. Therefore, our hypothesis that the most economical subjects in one exercise mode also have the best economy in another mode was confirmed especially for the skiing techniques.

From the error of measurement in our economy and the between-subject standard deviation in absolute values for oxygen uptake during the submaximal loads, we calculated the intraclass correlation to be 0.91 (Hopkins, 2006). This is close to the upper confidence limit for the correlation between the two skiing techniques (0.94). Hence, we cannot exclude the possibility that no other factors than common factors between the two techniques will determine the economy. These common factors could be similarities in technical elements between the techniques or intrinsic factors related to anthropometry or muscle fiber types. Double poling and V2-skating include similar poling actions with the arms and these poling actions produce most of the propulsive forces in both technics (Holmberg et al., 2005; Smith et al., 2009; Myklebust et al., 2013). Even if the leg work differs in the way that in double poling both legs work simultaneously while in skating the legs work alternatively (Holmberg et al., 2005; Myklebust et al., 2013), the legs movements in the two techniques are similar concerning ankle, knee, and hip extension and flexion cycles. Hence, one reason for the large correlation for exercise economy in the two techniques could be that because of the technical similarities, the skilled performer in one technique is also a skilled performer in the other. Therefore we cannot discriminate if technical elements or intrinsic factor are the main determinant for exercise economy in double poling and V2-skating.

To better discriminate between technique and intrinsic factor we also correlate running and skiing economy. Obviously skiing with the use of poles and skies were most of the propulsive force is transferred via the poles, is technically very different from running where all the propulsive forces is transferred to the ground via the legs. However, despite the use of the arms for propulsion in skiing, the legs are the biggest energy-consumer even during double poling (Calbet et al., 2005; Rud et al., 2013). Furthermore, both running and skiing are cyclic movements, with rhythmic flexion and extension in angle, knee and hip joints. Hence, if limb length and mass and/or fiber types were determinants for exercise economy, we would expect that the correlation between running and skiing economy to be large. The correlations between running and the skiing techniques were large, but lower than in between the skiing techniques with the upper confidence limit being 0.86. This is substantially less than the intraclass correlation of 0.91 (Hopkins, 2006). Hence, it is likely that other factors than intrinsic factors determine exercise economy. We believe that the other factors are related to technique and as mentioned, that the level of technical skills within each skier differs more between running and the skiing techniques, than between skiing techniques.

Interestingly, by excluding one subject from the analysis, the correlation between exercise economy in V2-skating and running increased from *r* = 0.53 to *r* = 0.82 and between double poling and running from *r* = 0.58 to *r* = 0.76. Notably, this “outlayer” (Figure 2) was also among the slowest skier during the 1000-m tests, scored low on technique abilities evaluated by expert skiing coaches, but in contrast, performed well in running events. Thus, this outlayer has probably a great potential to improve skiing economy during V2-skating and double poling and thereby increase skiing performance. We suggest that this can be done by improving the skiing technique.

In skiing, the external work can be determined by the inclination, speed and roller resistance of the skis. The energy turnover can be determined from the VO_2_ and the energy equivalent for oxygen corrected for the ratio between fat and carbohydrate combustion, which is estimated from RER values. Hence skiing gross efficiency can be calculated and the values for V2-skating and double poling were comparable (~16%) with values found by others (Sandbakk et al., 2010; Leirdal et al., 2013; Lindinger and Holmberg, 2011). Not surprisingly, there was an almost perfect correlation between exercise economy and gross efficiency in both skiing techniques since the oxygen equivalent normally varies only 1–3%. Hence, in this type of study, exercise economy can be used directly without effecting the conclusions.

### Cycle rate, anthropometrics, and economy

In running, it is generally accepted that athletes tend to automatically select their most economical cycle rate (Cavanagh and Williams, 1982). Our results support this observation, since there was only a moderate correlation between running cycle rate and economy. Our findings also indicate that athletes naturally select their most economical cycle rate in more technically demanding activities such as double poling and V2-skating since the correlation of exercise economy with cycle rate was trivial for V2-skating (*r* =−0.03; 90% confidence interval −0.53–0.57) and moderate for double poling (*r* = −0.46; −0.12–0.81). The finding is consistent with previous studies examining cycle rate in V2-alternate (Millet et al., 1998), V2-skating and V1-skating rollerskiing (Leirdal et al., 2013; Losnegard et al., 2012), and double poling (Lindinger and Holmberg, 2011). These findings indicate that cycle rate is not likely to be a major factor for determining exercise economy among highly trained cross-country skiers in ski skating, but in double pooling.

### Implications

The speed achieved in endurance competitions depends on several physiological and mechanical factors where one of these factors is the exercise economy. Hence, the sources of inter-individual variance are important to identify. The present study indicates that intrinsic factors limit the possibility for an individual athlete to improve exercise economy. It may be hypothesized, that the optimal exercise economy can be determine in an exercise mode were the athletes is highly skilled. For most athletes this may be during running. Hence, any deviation from a determined regression line between for instance running and skiing may indicate a potential for improvement in the skiing technique.

## Conclusion

The results indicate a very large correlation between the skiing techniques, and therefore that common intrinsic factors are important in determining exercise economy. This is supported by the fact that also a large correlation was found between the skiing techniques and running, and it is therefore highly likely that some common intrinsic factors determine both running and skiing economy. However, between skiing and running it is likely that also other factors are important for instance technical skills.

### Conflict of interest statement

The authors declare that the research was conducted in the absence of any commercial or financial relationships that could be construed as a potential conflict of interest.
